# Chronic rhinitis in glassblowers

**DOI:** 10.1186/2045-7022-3-S2-P10

**Published:** 2013-07-16

**Authors:** Nenad Baletic, Aleksandar Peric

**Affiliations:** 1 Military Medical Academy, Belgrade, Serbia; 2 Military Medical Academy, ENT, Belgrade, Serbia

## Introduction

At their workplace glassblowers are exposed to intensive infrared radiation, high temperature and low humidity of the air, hot gases, evaporations and dust particles, while the glassblower's pipe are the most important forms of exposure to noxious agents.

## The aim

1. To examine the prevalence of chronic rhinitis among glassblowers (experimental group) and control group based on objective findings at nasal mucosa. 2. To examine using appropriate statistical methods whether or not chronic rhinitis have higher prevalence in group of glassblowers. 3. To examine whether or not prevalence of chronic rhinitis depends on duration of exposure to noxious factors.

## Material and methods

Only noninfective chronic inflammation of nasal mucosa was considered in the present study. Infective, allergic, autonomic, eosinophylic, hormonal, autoimmune rhinitis, rhinitis "medicamentosa", as well as septal deviations or other anatomical deformities were excluded using appropriate standard diagnostic methods. For assessment of difference of chronic rhinitis prevalence in experimental and control group, we used chi-square test.

## Results

For comparison of chronic rhinitis prevalence in experimental and control group using chi-square test we got result: CHI=27.449, DF=2, P=1.614x10-7 <0.05; there was highly significant statistical difference between chronic rhinitis prevalence in experimental and control group. Remarkable graph of distribution of chronic rhinitis among glassblowers and control group was obtained in this study.

## Conclusion

Glassblowers have higher prevalence of chronic rhinitis than control group. Both factors, duration of exposure and membership of glassblowers cohort are important factors for chronic rhinitis, but membership of glassblowers cohort si more important factor.

